# SNPs in FNDC5 (irisin) are associated with obesity and modulation of glucose and lipid metabolism in Saudi subjects

**DOI:** 10.1186/s12944-016-0224-5

**Published:** 2016-03-11

**Authors:** Nasser M. Al-Daghri, Abdul Khader Mohammed, Omar S. Al-Attas, Osama E. Amer, Mario Clerici, Amal Alenad, Majed S. Alokail

**Affiliations:** Prince Mutaib Chair for Biomarkers of Osteoporosis, Biochemistry Department, College of Science, King Saud University, PO Box, 2455, Riyadh, 11451 Saudi Arabia; Biomarkers Research Program, Biochemistry Department, College of Science, King Saud University, Riyadh, 11451 Saudi Arabia; Department of Physiopathology and Transplantation, University of Milan, Milan, 20090 Italy; School of Biological Sciences, University of Southampton, Southampton, SO17 1BJ UK

**Keywords:** Obesity, Type 2 diabetes, Irisin, Triglyceride, Insulin, SNPs

## Abstract

**Background:**

Irisin is a recently identified myokine that plays an important role in preventing obesity and insulin resistance. We investigated whether the common FNDC5 (irisin precursor) gene variants influence susceptibility to obesity and type 2 diabetes (T2D) and verified the impact of FNDC5 gene variants on serum irisin levels, glucose and lipid metabolism in a Saudi population.

**Methods:**

Genomic DNA from 814 (394 T2DM and 414 controls) subjects were genotyped for the five common SNPs (rs3480A/G, rs1746661G/T, rs1298190A/G, rs726344A/G and rs1570569G/T) of the *FNDC5* gene using the TaqMan genotyping assay. Biochemical parameters and hematic concentrations of irisin and insulin as well as anthropometric indices were collected.

**Results:**

Serum irisin levels were higher in T2DM patients compared to controls (*p* < 0.0001). Analyses of FNDC5 SNPs showed that: 1) The rs3480 GG associates with decreased risk of obesity (*p* = 0.005; odds ratio: 0.48) and lower body mass index (BMI) values (*p* = 0.03). In addition, GGAAG was identified as the protective haplotype against risk of obesity (p = 0.001; odds ratio: 0.23). 2) The rs1746661 G allele associates with higher triglyceride (TG) levels (*p* = 0.019). 3) The rs157069 TT genotype associates with higher fasting insulin (*p* = 0.029) and HOMA-IR (*p* = 0.002) as well as with lower circulating irisin levels (*p* = 0.016).

**Conclusions:**

SNPs in FNDC5 gene correlates with obesity and glucose-lipid metabolism possibly because they modulate the serum levels of irisin.

**Electronic supplementary material:**

The online version of this article (doi:10.1186/s12944-016-0224-5) contains supplementary material, which is available to authorized users.

## Background

Obesity and insulin resistance play important roles in the pathogenesis of type 2 diabetes mellitus (T2DM) and are associated with cardiovascular disease [1, 2]. Imbalance between energy intake and energy expenditure may cause individuals to be underweight, overweight, or obese [3, 4]. Both skeletal muscle and adipose tissue have been shown to function as endocrine organs by secreting hormones called myokines and adipokines, respectively, and the cross-talk between them is critical for body weight and metabolism [5]. Adipose tissue is composed of two distinct compartments; white adipose tissue (WAT) and brown adipose tissue (BAT). WAT is involved in the regulation of energy homeostasis through the storage of excess energy, whereas BAT exert a thermogenic activity, and regulates body temperature by dissipating energy through heat production [6, 7]. Thus, browning of adipose tissue is hypothesized to improve insulin sensitivity and decrease weight gain [4].

Irisin, a newly described exercise-mediated myokine regulates energy metabolism by converting white into brown fat, may contribute to muscle-adipose tissue cross-talk [5, 8]. Irisin is produced upon cleavage of the precursor plasma membrane protein fibronectin type III domain-containing protein 5 (FNDC5) and enters the circulation [9]. In rodents, overexpression of adenoviral FNDC5 in high-fat diet fed mice results in increased energy expenditure, improved obesity (reduced body weight) and insulin resistance [10]. Furthermore, irisin is reported to be involved in the pathogenesis of several complications of obesity including dyslipidemia, T2DM, arterial hypertension and metabolic Syndrome [11]. However, there are discrepancies regarding the relation of irisin especially with obesity and T2DM. In some studies, a positive association was detected between irisin levels and BMI [9, 12, 13], while others reported null [14, 15] or a negative correlation [11, 12, 16]. From the above mentioned studies, it is evident that either irisin or its precursor (FNDC5) has a role to play in pathogenesis of metabolic diseases, including diabetes and obesity.

Until now, only three previous genetic studies have investigated the association of common SNPs in FNDC5 with obesity [17], insulin sensitivity [18] and glucose metabolism [19]. Because of the pivotal role of irisin/FNDC5 in obesity, insulin resistance and T2DM, we aim to evaluate a possible role of five *FNDC5* polymorphisms (rs3480A/G, rs1746661G/T, rs1298190A/G, rs726344A/G and rs1570569G/T) in obesity and T2DM phenotypes from Saudi Arabia, a region with a high prevalence of both conditions [20, 21]. In addition, we examined whether FNDC5 gene variants has any influence on serum irisin expression and clinical traits related to glucose and lipid metabolism.

## Results

The anthropometric, clinical and biochemical features of individuals enrolled in the study are presented in Table 1. All the analyzed SNPs (rs3480, rs1746661, rs1570569 and rs1298190) were in Hardy-Weinberg equilibrium in our population (*p* > 0.05) except rs726344. The role of these common *FNDC5* gene variants in predisposition to T2DM was analyzed next by multiple logistic regression model using age, gender, and BMI as covariates. Results indicated that none of these SNPs were associated with T2DM (Table 2). We then analyzed the distribution of these SNPs in two BMI-based subgroups, namely, obese or lean. Logistic regression indicated that the rs3480 GG genotype is protective against obesity (*p* = 0.005; OR, 0.48; 95 % CI, 0.28-0.79) (Table 3). This was confirmed by a significant association of the same GG genotype of rs3480 with lower BMI values (*p* = 0.03) (Table 4). In addition, the distribution frequencies of rs3480 _G + rs1746661_G + rs1298190 _A + rs726344 _A + rs1570569_G (i.e. GGAAG) haplotype was significantly different in obese compared to lean individuals; notably, this haplotype is significantly associated with reduced obesity risk (*p* = 0.001; OR, 0.23; 95 % CI, 0.10–0.53) (Table 5). However, no such differences were observed in T2DM group for any of the examined haplotypes (Table 5).

**Table 1:** Anthropometric and metabolic characteristics of subjects

	Group		Sub-group	
Variables	T2DM	Non-T2DM	Obese	Lean
	(*N* = 394)	(*N* = 420)	(*N* = 293)	(*N* = 264)
Male/Female	234/160	188/232	121/172	150/114
Age (years)	53.5 ± 11.2	44.3 ± 12.7**	47.8 ± 11.12	48.4 ± 14.9
BMI (kg/m2)	29.5 ± 6.0	27.2 ± 5.3**	34.9 ± 4.39	21.7 ± 2.8**
Systolic BP (mmHg)	128.8 ± 15.1	117 ± 14.0**	125.4 ± 14.2	119 ± 14.3**
Diastolic BP (mmHg)	80.6 ± 9.2	77.1 ± 10.1**	79.7 ± 10.2	75.4 ± 7.7**
Total Cholesterol (mmol/l)	5.3 ± 1.17	5.0 ± 1.17**	5.18 ± 1.1	5.17 ± 1.2
Triglycerides (mmol/l)	1.7 ± 0.6	1.3 ± 0.56**	1.5 ± 0.57	1.45 ± 0.61*
Glucose (mmol/l)	10.1 ± 3.59	5.1 ± 0.93**	10.1 ± 3.59	5.1 ± 0.93*
HDL-Cholesterol (mmol/)	0.85 ± 0.34	0.92 ± 0.40*	0.90 ± 0.37	0.87 ± 0.39
LDL-Cholesterol (mmol/)	3.63 ± 1.0	3.56 ± 1.0	3.60 ± 1.0	3.56 ± 1.0
Insulin (μIU/mL)	15.08 ± 9.0	15.9 ± 9.1	17.6 ± 9.4	10.6 ± 6.3**
HOMA-IR	6.88 ± 5.5	3.7 ± 2.35**	6.35 ± 4.4	3.0 ± 2.1**

*Note*: “**” denotes *p* < 0.001, “*” represents *p* < 0.01. Independent sample t-test was used to compute the difference between studied groups. Data were presented as mean ± standard deviation. Insulin and HOMA-IR are log transformed prior to analysis. Insulin and HOMA-IR values were available for 392 subjects

**Table 2:** Genotype distribution in T2DM vs Non-T2DM subjects

	T2DM (%)	Non-T2DM (%)	Odds ratio (95 CI)	*P*-value
rs3480 A/G				
GG	78 (20.7)	88 (21.5)	1.29 (0.82–2.03)	0.26
AG	181 (48.1)	186 (45.4)	1.34 (0.93–1.94)	0.11
AA	117 (31.1)	136 (32.2)	**1.0**	**1.0**
rs1746661 G/T				
TT	27 (7.2)	25 (6.8)	1.22 (0.64–2.35)	0.53
GT	125 (33.5)	113 (30.9)	1.16 (0.81–1.67)	0.40
GG	221 (59.2)	228 (62.3)	**1.0**	**1.0**
rs1298190 A/G				
GG	7 (1.8)	10 (2.4)	0.54 (0.16–1.76)	0.31
AG	85 (22.4)	90 (22)	1.10 (0.75–1.63)	0.60
AA	288 (75.8)	309 (75.6)	**1.0**	**1.0**
rs7246334 A/G				
GG	2 (0.5)	1 (0.3)	–	–
AG	280 (75.3)	290 (74.7)	–	–
AA	90 (24.2)	97 (25)	–	–
rs1570569 G/T				
TT	25 (6.6)	23 (5.6)	1.45 (0.72–2.8)	0.28
GT	132 (34.7)	139 (33.9)	1.08 (0.76–1.52	0.66
GG	223 (58.7)	248 (60.5)	**1.0**	**1.0**

*Note*: The genotype distribution of rs3480 (A/G), rs1746661 (G/T), rs1298190 (A/G) rs7246344 (A/G) and rs1570569 (G/T) variants of FNDC5 gene. Genotype frequency differences between T2DM and Non-T2DM were tested for each SNP using Chi-square test. Odds ratios (ORs) and 95 % confidence intervals for genotypes were calculated using multinomial logistic regression analyses considering T2DM as dependent variable adjusted for age, gender and BMI. The most common genotype was used as the reference

Bold means reference value

**Table 3:** Genotype distribution in obese vs Non-obese subjects

	Obese (%)	Non-obese (%)	Odds ratio (95 CI)	*P*-*value*
rs3480 A/G				
GG	49 (17.2)	66 (25.8)	0.48 (0.28–0.79)	0.005
AG	136 (47.7)	117 (45.7)	0.76 (0.50–1.15)	0.20
AA	100 (35.1)	73 (28.5)	**1.0**	**1.0**
rs1746661 G/T				
TT	22 (8.7)	18 (7.0)	1.30 (0.64–2.6)	0.47
GT	83 (32.7)	83 (32.4)	1.04 (0.69–1.56)	0.82
GG	149 (59.7)	155 (60.5)	**1.0**	**1.0**
rs1298190 A/G				
GG	5 (1.8)	3 (1.2)	1.25 (0.26–6.0)	0.77
AG	64 (22.5)	63 (24.5)	0.88 (0.58–1.35)	0.57
AA	216 (75.8	191 (74.3)	**1.0**	**1.0**
rs1570569 G/T				
TT	19 (6.6)	13 (5.1)	1.33 (0.60–2.92)	0.47
GT	100 (35)	89 (34.8)	1.06 (0.72–1.56)	0.74
GG	167 (58.4)	154 (60.2)	**1.0**	**1.0**

*Note*: The genotype distribution of rs3480 (A/G), rs1746661 (G/T), rs1298190 (A/G) and rs1570569 (G/T) variants of FNDC5 gene. Genotype frequency differences between obese and non-obese were tested for each SNP using Chi-square test. Odds ratios (ORs) and 95 % confidence intervals for genotypes were calculated using multinomial logistic regression analyses using obesity as dependent variable adjusted for age, gender and T2DM. Significance was set at *p* < 0.05. The most common genotype was used as the reference. The variant rs7246344 (A/G) was excluded from analysis due to low rare genotype frequency

Bold means reference value

**Table 4:** Differences in various metabolic parameters according to FNDC5 gene SNPs

	rs3480 A/G				rs1746661 G/T			
Variables	GG	AG	AA	P	TT	GT	GG	P
BMI (kg/m^2^)	27.2 ± 6.4	28.3 ± 6.3	29.6 ± 6.5	0.030*	27.9 ± 6.6	28.2 ± 6.8	28.0 ± 6.1	0.93
Insulin (μIU/mL)#	13.54 ± 7.5	16.1 ± 9.5	15.8 ± 9.2	0.24	16.0 ± 8.4	14.8 ± 8.4	15.6 ± 9.6	0.70
HOMA-IR #	4.8 ± 3.9	5.7 ± 4.6	5.2 ± 3.9	0.17	5.8 ± 4.0	5.1 ± 3.6	5.4 ± 4.6	0.64
Glucose (mmol/l)	7.5 ± 3.5	7.3 ± 3.4	7.2 ± 3.4	0.78	7.8 ± 3.5	7.4 ± 3.4	7.4 ± 3.6	0.92
Total Cholesterol (mmol/l)	5.1 ± 1.1	5.1 ± 1.1	5.3 1 ± 1.1	0.66	5.2 ± 1.1	5.2 ± 1.2	5.2 ± 1.1	0.90
HDL-Cholesterol (mmol/l)	0.87 ± 0.3	0.84 ± 0.3	0.94 ± 0.4‡	**0.015**	0.89 ± 0.3	0.85 ± 0.3	0.89 ± 0.3	0.20
Triglycerides (mmol/l)	1.45 ± 0.6	1.55 ± 0.5	1.55 ± 0.58	0.13	1.31 ± 0.5	1.58 ± 0.6	1.53 ± 0.5	0.019*
LDL-Cholesterol (mmol/l)	3.60 ± 0.9	3.55 ± 1.0	3.61 ± 1.0	0.80	3.66 ± 0.8	3.60 ± 1.1	3.57 ± 0.9	0.23
Irisin (μg/ml)#	0.70 ± 0.6	0.63 ± 0.5	0.77 ± 0.7	0.62	0.50 ± 0.3	0.77 ± 0.5	0.89 ± 0.7	0.10
	rs1570569 G/T				rs1298190 A/G			
Variables	TT	GT	GG	P	GG	AG	AA	P
BMI (kg/m^2^) (mmol/l)	28.4 ± 6.4	28.4 ± 6.8	28.3 ± 6.1	0.94	28.3 ± 4.7	27.9 ± 6.2	28.4 ± 6.5	0.62
Insulin (μIU/mL)#	21.1 ± 11.5‡	14.6 ± 8.2	15.5 ± 9.3	0.029*	14.9 ± 10.1	14.6 ± 8.8	15.8 ± 9.2	0.74
HOMA-IR#	9.5 ± 7.4‡	5.0 ± 3.6	5.2 ± 4.1	0.002*	3.7 ± 2.1	5.4 ± 4.6	5.4 ± 4.1	0.56
Glucose (mmol/l)	8.0 ± 3.9	7.3 ± 3.3	7.3 ± 3.5	0.93	6.7 ± 2.7	7.7 ± 3.8	7.2 ± 3.4	0.40
Total cholesterol (mmol/l)	5.2 ± 1.2	5.1 ± 1.2	5.2 ± 1.1	0.73	4.8 ± 0.7	5.2 ± 1.0	5.1 ± 1.2	0.54
HDL-Cholesterol (mmol/l)	0.86 ± 0.4	0.84 ± 0 .3	0.90 ± 0.35	0.08	0.85 ± 0.2	0.85 ± 0.2	0.89 ± 0.3	0.67
Triglycerides (mmol/l)	1.44 ± 0.6	1.54 ± 0.6	1.53 ± 0.57	0.54	1.52 ± 0.58	1.51 ± 0.6	1.55 ± 0.5	0.77
LDL-Cholesterol (mmol/l) (mmol/l)	3.66 ± 1.0	3.60 ± 1.1	3.5 ± 0.98	0.85	3.48 ± 0.52	3.57 ± 0.9	3.58 ± 1.0	0.93
Irisin (μg/ml) #	0.41 ± 0.3	0.71 ± 0.6	0.72 ± 0.6	0.016*	0.90 ± 0.6	0.64 ± 0.6	0.70 ± 0.6	0.54

*Note*: Data were presented as mean ± SD values. #log transformed prior to analysis. Univariate general linear model adjusted for appropriate covariates such as age, gender, BMI and T2DM. “*” indicates significance after Bonferroni correction; “‡” indicates significantly different from both the genotypes. Significance was set at *p* < 0.05

Bold means significant

**Table 5:** Haplotype frequency of FNDC5 variants in T2DM versus non-T2DM and obese versus lean subjects

Haplotype	T2DM	Control	Odd ratio (95 % CI)	*P*-value	Obese	Lean	Odd ratio (95 % CI)	*P*-value
AGAAG	119	125	1.00 (Reference)	–	95	73	1.00 (Reference)	–
AGAGG	87	99	0.93 (0.63–1.35)	0.681	65	61	0.82 (0.52–1.30)	0.398
GGAAG	27	28	1.01 (0.56–1.82)	0.966	8	27	0.23 (0.10–0.53)	**0.001**
GGAGG	9	14	0.68 (0.28–1.62)	0.379	11	6	1.41 (0.50–3.99)	0.519
GGGAG	45	50	0.95 (0.59–1.52)	0.817	32	32	0.77 (0.43–1.37)	0.371
GTAAT	39	45	0.91 (0.55–1.50)	0.711	33	27	0.94 (0.52–1.70)	0.836
GTAGT	47	39	1.27 (0.77–2.07)	0.349	29	30	0.74 (0.41–1.35)	0.327
GTGAG	3	5	0.63 (0.15–2.69)	0.533	4	3	1.02 (0.22–4.72)	0.975

*Note*: Order of SNPs: rs3480A/G, rs1746661G/T, rs1298190A/G, rs726344A/G and rs1570569G/T). Pearson’s p value and OR (95 % CI) were calculated by haplotype analysis

Bold means significant

To determine the effect of *FNDC5* SNPs on clinical and biochemical parameters, we analyzed the distribution of these variables in relation to the various genotypes. After controlling for age, gender, BMI and T2DM, we observed that the rs1746661 G allele was significantly associated with higher triglycerides (TG) levels (*p* = 0.019) (Table 4). Similarly, the rs3480 AA genotype was associated with higher HDL-Cholesterol levels. Finally, the rs1570569 TT genotype was significantly associated with higher fasting serum insulin (*p* = 0.029) and HOMA-IR values (*p* = 0.002) (Table 4). Importantly, all the associations remained significant after Bonferroni correction.

**Fig. 1:**
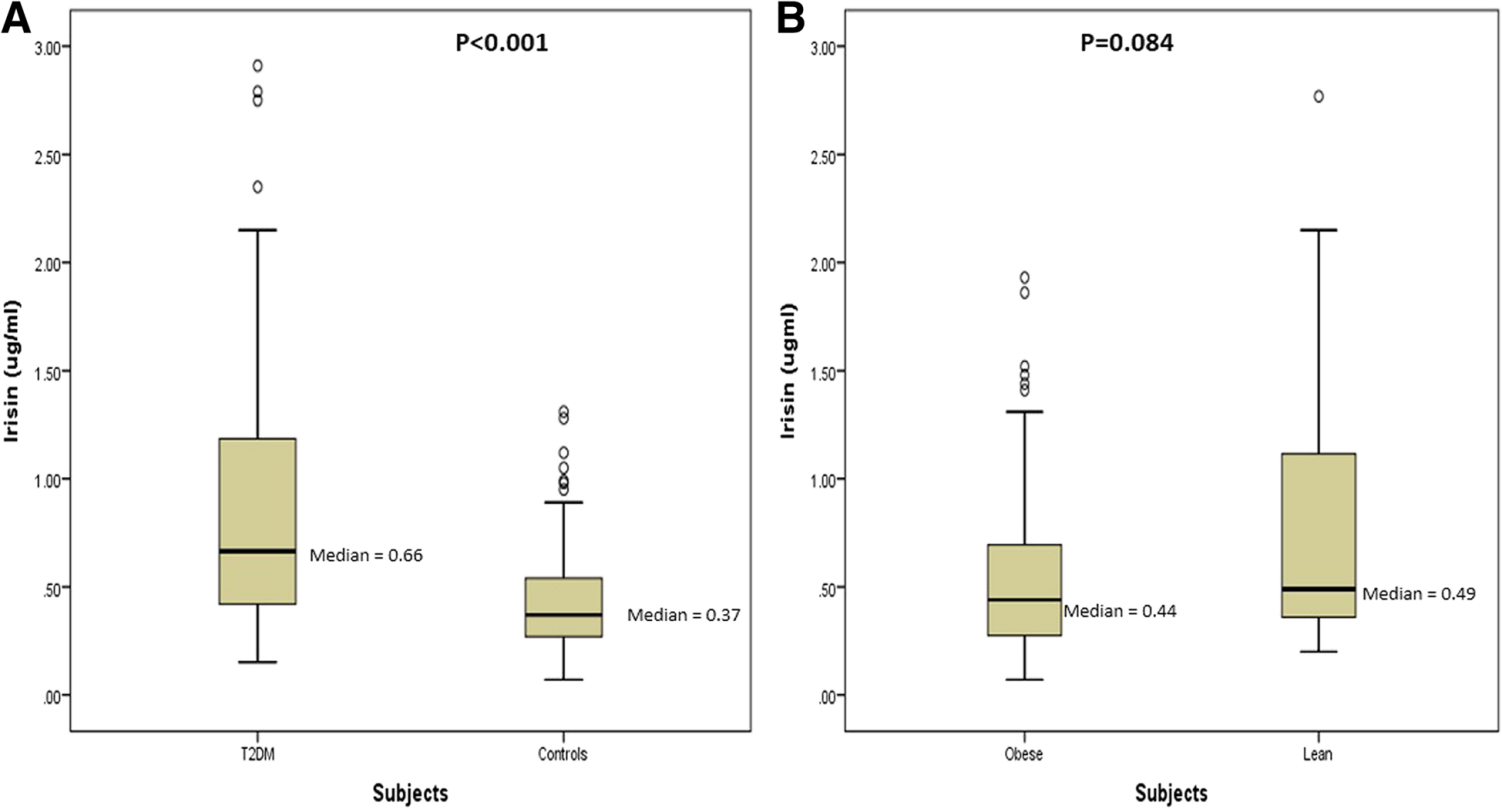
**a** Median circulating irisin levels in T2DM vs Non-T2DM group. **b** Median circulating irisin levels in obese vs lean group. Results are shown as median values for each group

Serum irisin levels were analyzed in a randomly selected subgroup of 88 T2DM and 119 controls subjects. Significantly higher circulating irisin levels were observed in the T2DM than in non-T2DM subjects (*p* < 0.001) (Fig. 1a), whereas, no such differences were observed in obese and lean group (Fig. 1b). Assessment of possible associations between *FNDC5* SNPs and serum irisin levels after adjusting age, gender, BMI and T2DM showed that the *FNDC5* rs1570569 TT genotype was associated with lower serum irisin levels (*p* = 0.016) (Table 4).

## Discussion

In the attempt to shed light on the relationship between irisin (FNDC5 gene), T2DM and obesity, we performed genetic analysis (SNP) on a cohort of 394 T2D and 293 obese individuals, comparing the results to those obtained in healthy controls. Results showed that rs3480, an intronic variant in FNDC5 gene, is protective against obesity. Additional data indicated that higher circulating irisin levels were found in T2DM patients, and that variants of the FNDC5 gene modulate circulating irisin levels and glucose-lipid profile.

Irisin, a novel myokine expressed in skeletal muscle is stimulated by exercise. This hormone exerts beneficial effects on metabolism by inducing the browning of subcutaneous white adipocytes and contributes to the organ crosstalk, which is essential to maintain metabolic homeostasis [22, 23]. Both in culture and mouse models, FNDC5 induces browning of subcutaneous adipocytes and thermogenesis by increasing uncoupling protein 1 (UCP1) levels [10]. Serum irisin has been linked to a variety of disorders, including obesity, insulin sensitivity and T2DM [12, 18, 24]. Furthermore, it has also been reported that the SNPs in FNDC5 modulates glucose metabolism and insulin sensitivity [17–19]. To the best of our knowledge, the relationship between variants of FNDC5 (Irisin) and its association with T2DM and obesity phenotypes in the Saudi Arabian population has not been previously studied. Remarkably, results of our study showed the presence of a strong association between the FNDC5 rs3480 (GG) genotype and a reduced risk (protection) of obesity, independent of age, gender and T2DM. A confirmation of this finding was obtained by analysis of BMI values, which were lower in subjects carrying the alternative homozygous GG genotype. However, the exact molecular mechanism and reasons underlying these associations remain to be elucidated.

Peroxisome proliferator-activated receptor alpha (PPAR-α), a chief transcriptional regulator of lipid metabolism, is upregulated in adipose cells treated with FNDC5 [10]. PPAR-α upregulates the gene encoding fibroblast growth factor 21 (FGF21), expressed within the liver, and enhances hepatic lipid oxidation and blood TG clearance [19]. It has also been reported that circulating irisin levels are associated with serum TG, LDL-C and total cholesterol in patients with insulin-resistant states. [25], HDL-C in patients with chronic kidney disease [26], with FBG and HOMA-IR in children [22], suggesting a possible role of irisin and its precursor FNDC5 in regulation of lipid and glucose metabolism. Thus, we examined the association between these metabolic parameters, known to be altered in T2DM and obesity, and FNDC5 gene variants. Results showed that the G allele of rs3480 is associated with low HDL-C levels. In contrast, Staiger et al. [18] and Tanisawa et al. [19] did not detect any association between rs3480 and HDL-C. However, Tang et al. [17] have reported that rs3480 has a trend towards association with HDL-C and LDL-C levels in overweight subjects.

In the present study, we report that the rs1570569 TT genotype is associated with increased fasting serum insulin levels and HOMA-IR values. In contrast, Tanisawa et al. [19] demonstrated that the G allele of rs3480 correlates with high HOMA-IR levels in Japanese men. A study by Tang et al. [17] reported the effect rs16835198 on fasting insulin was significantly modified by BMI in the Chinese Han population. Further, a study by Staiger et al. [18] reported SNP rs16835198 and rs726344 effects on parameters of insulin sensitivity. However, in our study, rs726344 was excluded from statistical analyses due to its extremely low rare genotype frequency.

Contrary to our expectation, we detected higher serum irisin levels in T2DM patients than non-diabetic subjects. However, most of the previous studies in humans reported lower irisin levels in T2DM patients [12, 16, 24, 27], although these findings were contradicted by other investigations [11, 28]. Recently, Kurdiova et al. [28] reported that myotubes derived from T2DM patients showed higher *FNDC5* expression and displayed higher irisin levels media compared to that of lean donors. In addition, Park et al. [11] also concluded that irisin is associated with increased odds of having metabolic syndrome and insulin resistance, suggesting physiological compensatory mechanism that would result in increased irisin levels in metabolic diseases due to an underlying decreased sensitivity to irisin’s effects.

Although one study previously reported that two common SNPs (rs3480 and rs16835198) in FNDC5 locus are not associated with serum irisin levels [19]. However, the results from our study indicate that the subjects with the rare rs1570569 (TT) genotype had significantly lower circulating irisin levels. The relation we detected between rs1570569 variant and serum low irisin, high insulin, and HOMA-IR levels suggest that this particular SNP in the irisin precursor gene may affect glucose metabolism through altering circulating irisin levels. Because this SNP is located in the intronic region of FNDC5 gene, this allelic variant may not directly influence the amino acid sequence of the irisin product. However, it should be noted that introns harbor functional polymorphisms that can influence the expression of the genes that host them by altering mRNA stability, alternative mRNA splicing, or the binding of transcription factors [29, 30]. Moreover, Sanchis-Gomar et al. [30] reported that the SNP (rs726344) in FNDC5 gene had functional significance as the variant A-allele having higher luciferase activity compared with the G-allele.

The reason for discrepancies in association of the above mentioned SNPs with serum lipids, insulin, HOMA-IR and irisin can be partially explained by fact that the effects of SNPs on health-related phenotypes are not consistent among diverse ethnic groups due to differences in genetic backgrounds and allelic frequencies. Thus, further studies with larger sample sizes are needed to confirm these associations.

## Conclusions

In summary, our data suggest that none of the five analyzed SNPs in FNDC5 gene showed association with T2DM in Saudi population. However, the SNP rs3480 and GGAAG haplotype are associated with reduced risk of obesity. In addition, rs1746661 variant is associated with elevated triglycerides; rs157069 is associated with increased serum insulin and HOMA-IR values as well as decreased circulating irisin levels. Further analyses will be required to gain insight into the underlying mechanisms.

## Methods

### Patients and controls

A total of 814 unrelated adult Saudi individuals [394 confirmed T2DM (234 males and 160 females) and 420 controls (188 males and 232 females] were randomly selected from the Biomarker Screening Project in Riyadh (RIYADH COHORT), a capital-wide epidemiological study of over 17,000 consenting Saudis coming from different Primary Health Care Centers (PHCCs) in Kingdom of Saudi Arabia (KSA). Diagnosis of T2DM was based on the World Health Organization (WHO) proposed cut-off (fasting serum glucose ≥7.0 mmol/l or 126 mg/dl). Subjects with comorbidities that needed medical attention or with medical complications (coronary artery disease, nephropathy, thyroid diseases and end stage renal or liver disease) were excluded from the study. Using the WHO criteria, BMI was used to classify the studied individuals into two sub-groups, that is, normal weight (BMI <25 kg/m^2^), and obese (BMI ≥30 kg/m^2^). Both control and T2DM patients were included in these sub-groups. The clinical characteristics of all participants are shown in Table 1. Written informed consent was collected before inclusion in the study and ethics approval was granted by the Ethics Committee of the College of Science, King Saud University, Riyadh, KSA. Subjects were recruited from their homes using a random cluster sampling strategy. Consenting participants were then requested to visit the nearest participating PHCC for questionnaire administration, anthropometric measurement and blood extraction. A questionnaire focusing on demographic information and past and present medical history was given to all subjects.

### Anthropometry and blood collection

Subjects were requested to visit their respective PHCCs following overnight fasting (>10 h) for anthropometry and blood withdrawal. Anthropometry included height (rounded off to the nearest 0.5 cm), weight (rounded off to the nearest 0.1 kg), waist and hip circumference (centimeters), and mean systolic and diastolic blood pressure (millimeters of Hg) (average of 2 readings). Body mass index (BMI) was calculated as weight in kilograms divided by height in square meters. Fasting blood samples were collected and transferred immediately to a non-heparinized tube for centrifugation. Collected serum was then transferred to pre-labeled plain tubes, stored in ice and delivered to the Biomarkers Research Program (BRP) in King Saud University, Riyadh, KSA, for immediate storage at −20 °C.

### Biochemical analyses

Fasting glucose and lipid profile were measured using a chemical analyzer (Konelab, Espoo, Finland). Serum irisin and insulin was assessed using an enzyme-linked immunosorbent assay (ELISA) for with an intra-assay variability of <10 % and inter-assay variation of <15 %. All fasting samples fell within the detection range. Serum irisin and insulin were measured for 25 % and 50 % of the studied samples respectively. Quality Assurance (QA) standards are maintained by ISO 9000 and 17025, whereas the QA department audits the BRP laboratory at regular intervals. Insulin resistance index was estimated using the homeostasis model assessment (HOMA), which was calculated using fasting plasma glucose and insulin levels.

### Single nucleotide polymorphism (SNP) selection and genotyping

Based on publicly available NCBI SNP variation database [31] we selected five tagging SNPs with global minor allele frequency of < 0.05 (Additional file 1: Table S1) in a region encompassing the *FNDC5* gene and its 2 kb up and downstream sequences.

### Genotyping

Genomic DNA was isolated from whole blood using the blood genomicPrep mini spin kit (GE healthcare Life Sciences, Piscataway, NJ, USA). DNA concentration and purity (260/280) were checked using Nano-drop spectrophotometer. The five tagging SNPs (rs3480A/G, rs1746661G/T, rs1298190A/G, rs726344A/G and rs1570569G/T) were evaluated by allelic discrimination Real-time PCR using pre-designed TaqMan genotyping assays from Applied Bio-systems, Foster City, CA, USA. TaqMan assay IDs with context sequence (primers and probes) are presented in Additional file 1: Table S1. Amplification reactions were performed in a volume of 10 μL containing 1X TaqMan genotyping Master Mix (Applied Biosystems), 1X mix of unlabeled PCR primers and TaqMan MGB probes, and 30 ng of template DNA. All amplification and detection was conducted on genomic DNA in 96-well PCR plates using a Bio-Rad CFX96 Real-Time PCR Detection System (Bio-Rad, Milan, Italy). Thermal cycling was initiated with a denaturation step of 10 min at 95 °C, followed by 45 cycles of 15 s at 95 °C and 90 s at 60 °C. After PCR was completed, allelic discrimination was analyzed using the Bio-Rad CFX Manager Software (Version 1.6, Bio-Rad). Genotype assignment was determined by plotting the end point relative fluorescent units (RFU) for one fluorophore (allele 1 on the x-axis) against the RFU for the other fluorophore (allele 2 on the y-axis) on the allelic discrimination. All PCR reactions were set up in a dedicated PCR area with dedicated PCR pipettes and reagents. For validation, about thirty random samples were re-genotyped. The results were reproducible with no discrepancies in genotyping.

### Statistical analysis

Data were analyzed using SPSS version 21.0 (IBM, Armonk, NY, USA). Significance was set at *p* < 0.05. Biochemical parameters were expressed as mean ± standard deviation (SD). Hardy-Weinberg equilibrium was tested using Chi-square test. Before carrying out parametric statistical procedures, insulin, HOMA-IR and irisin levels were logarithmically transformed to ensure a normal distribution. Genotype distributions between the cases and control subjects were compared with Chi-square test. Odds ratios (ORs) and 95 % confidence intervals (CIs) were calculated by multinomial logistic regression using genotypes as the factor with sex and age as covariates. BMI was added as a covariate when addressing the association between T2DM and *FNDC5* variants; T2DM was accounted as covariate for addressing the effect of SNPs on obesity. Univariate general linear model adjusted for appropriate covariates such as age, BMI, gender and T2DM was used to compare different genotypes in each SNP with various parameters, followed by Bonferroni correction. Haplotype frequencies were estimated by Expectation–Maximization algorithm (EM algorithm) with haplostats using R statistical package [32]. The most common haplotype was used as the reference and rare haplotypes were dropped from the analysis.

## Additional file

Additional file 1: Table S1. Details of used TaqMan assays, chromosomal location and minor allele frequencies for the studied SNPs. (DOCX 13 kb)

## Abbreviations

BAT: brown adipose tissue
BMI: body mass index
CI: confidence intervals
ELISA: enzyme-linked immunosorbent assay
FNDC5: fibronectin type III domain-containing protein 5
HDL-C: HDL cholesterol
HOMA-IR: homeostasis model assessment for insulin resistance
LDL-C: LDL cholesterol
OR: odds ratio
PHCCs: primary health care centers
SNPs: single nucleotide polymorphisms
T2DM: type 2 diabetes mellitus
TG: triglycerides
WAT: white adipose tissue

## References

[1] Højlund K, Boström P (2012). Irisin in obesity and type 2 diabetes. J Diabetes Complications.

[2] Hotta K, Kitamoto A, Kitamoto T, Mizusawa S, Teranishi H, So R (2012). Replication study of 15 recently published Loci for body fat distribution in the Japanese population. J Atheroscler Thromb.

[3] Villarejo C, Fernández‐Aranda F, Jiménez‐Murcia S, Peñas‐Lledó E, Granero R, Penelo E (2012). Lifetime obesity in patients with eating disorders: increasing prevalence, clinical and personality correlates. Eur Eat Disord Rev.

[4] Al-Daghri NM, Al-Attas OS, Alokail MS, Alkharfy KM, Yousef M, Vinodson B, et al. Maternal inheritance of circulating irisin in humans. Clin Sci. 2014;126:837-44. 10.1042/CS20130426 24397868

[5] Reinehr T, Elfers C, Lass N, Roth CL (2015). Irisin and its relation to insulin resistance and puberty in obese children: a longitudinal analysis. J Clin Endocrinol Metab.

[6] Elsen M, Raschke S, Eckel J (2014). Browning of white fat: does irisin play a role in humans?. J Endocrinol.

[7] Beranger GE, Karbiener M, Barquissau V, Pisani DF, Scheideler M, Langin D (1831). In vitro brown and “brite”/“beige” adipogenesis: human cellular models and molecular aspects. Biochim Biophys Acta.

[8] Chen J-q, Huang Y-y, Gusdon AM, Qu S (2015). Irisin: a new molecular marker and target in metabolic disorder. Lipids Health Dis.

[9] Huh JY, Panagiotou G, Mougios V, Brinkoetter M, Vamvini MT, Schneider BE (2012). FNDC5 and irisin in humans: I. Predictors of circulating concentrations in serum and plasma and II. mRNA expression and circulating concentrations in response to weight loss and exercise. Metabolism.

[10] Boström P, Wu J, Jedrychowski MP, Korde A, Ye L, Lo JC (2012). A PGC1-[agr]-dependent myokine that drives brown-fat-like development of white fat and thermogenesis. Nature.

[11] Park KH, Zaichenko L, Brinkoetter M, Thakkar B, Sahin-Efe A, Joung KE (2013). Circulating Irisin in relation to insulin resistance and the metabolic syndrome. J Clin Endocrinol Metab.

[12] Stengel A, Hofmann T, Goebel-Stengel M, Elbelt U, Kobelt P, Klapp BF (2013). Circulating levels of irisin in patients with anorexia nervosa and different stages of obesity–Correlation with body mass index. Peptides.

[13] Pardo M, Crujeiras AB, Amil M, Aguera Z, Jiménez-Murcia S, Baños R et al. Association of irisin with fat mass, resting energy expenditure, and daily activity in conditions of extreme body mass index. Int J Endocrinol. 2014;2014:857270. 10.1155/2014/857270 PMC4016898 24864142

[14] Anastasilakis AD, Polyzos SA, Saridakis ZG, Kynigopoulos G, Skouvaklidou EC, Molyvas D (2014). Circulating irisin in healthy, young individuals: day-night rhythm, effects of food intake and exercise, and associations with gender, physical activity, diet, and body composition. J Clin Endocrinol Metab.

[15] Sanchis-Gomar F, Alis R, Pareja-Galeano H, Sola E, Victor VM, Rocha M (2014). Circulating irisin levels are not correlated with BMI, age, and other biological parameters in obese and diabetic patients. Endocrine.

[16] Moreno-Navarrete JM, Ortega F, Serrano M, Guerra E, Pardo G, Tinahones F (2013). Irisin is expressed and produced by human muscle and adipose tissue in association with obesity and insulin resistance. Clin Endocrinol Metab.

[17] Tang S, Zhang R, Jiang F, Wang J, Chen M, Peng D (2014). An Interaction between a FNDC5 Variant and Obesity Modulates Glucose Metabolism in a Chinese Han Population. PLoS One.

[18] Staiger H, Böhm A, Scheler M, Berti L, Machann J, Schick F (2013). Common genetic variation in the human FNDC5 locus, encoding the novel muscle-derived ‘browning’factor irisin, determines insulin sensitivity. PLoS One.

[19] Tanisawa K, Taniguchi H, Sun X, Ito T, Cao Z-B, Sakamoto S (2014). Common single nucleotide polymorphisms in the FNDC5 gene are associated with glucose metabolism but do not affect serum irisin levels in Japanese men with low fitness levels. Metabolism.

[20] Al-Daghri NM, Clerici M, Al-Attas O, Forni D, Alokail MS, Alkharfy KM (2013). A nonsense polymorphism (R392X) in TLR5 protects from obesity but predisposes to diabetes. J Immunol.

[21] Al-Daghri NM, Guerini FR, Al-Attas OS, Alokail MS, Alkharfy KM, Draz HM (2014). Vitamin D Receptor Gene Polymorphisms Are Associated with Obesity and Inflammosome Activity. PLoS One.

[22] Al‐Daghri NM, Alkharfy KM, Rahman S, Amer OE, Vinodson B, Sabico S (2014). Irisin as a predictor of glucose metabolism in children: sexually dimorphic effects. Eur J Clin Invest.

[23] Sesti G, Andreozzi F, Fiorentino T, Mannino G, Sciacqua A, Marini M, et al. High circulating irisin levels are associated with insulin resistance and vascular atherosclerosis in a cohort of nondiabetic adult subjects. Acta Diabetol. 2014;1–9. 10.1007/s00592-014-0576-0 24619655

[24] Liu J-J, Wong MD, Toy WC, Tan CS, Liu S, Ng XW (2013). Lower circulating irisin is associated with type 2 diabetes mellitus. J Diabetes Complications.

[25] Li M, Yang M, Zhou X, Fang X, Hu W, Zhu W, et al. Elevated circulating levels of irisin and the effect of metformin treatment in women with polycystic ovary syndrome. J Clin Endocrinol Metab. 2015;100:1485-93. 10.1210/jc.2014-2544 25675380

[26] Wen M-S, Wang C-Y, Lin S-L, Hung K-C (2013). Decrease in irisin in patients with chronic kidney disease. PLoS One.

[27] Choi Y-K, Kim M-K, Bae KH, Seo H, Jeong J-Y, Lee W-K (2013). Serum irisin levels in new-onset type 2 diabetes. Diabetes Res Clin Pract.

[28] Kurdiova T, Balaz M, Vician M, Maderova D, Vlcek M, Valkovic L (2014). Are Skeletal Muscle & Adipose Tissue Fndc5 Gene Expression and Irisin Release Affected by Obesity, Diabetes and Exercise? In vivo & in vitro studies. J Physiol.

[29] Cooper DN (2010). Functional intronic polymorphisms: buried treasure awaiting discovery within our genes. Hum Genomics.

[30] Sanchis-Gomar F, Garatachea N, He Z-h, Pareja-Galeano H, Fuku N, Tian Y (2014). FNDC5 (irisin) gene and exceptional longevity: a functional replication study with rs16835198 and rs726344 SNPs. Age.

[31] National Center for Biotechnology Information, U.S. National Library of Medicine 8600 Rockville Pike, Bethesda MD, 20894 USA. http://www.ncbi.nlm.nih.gov/variation/view/?q=FNDC5. Accessed 12 February 2014.

[32] R Development Core Team. R: A language and environment for statistical computing. R Foundation for Statistical Computing, Vienna, Austria. (http://www.R-project.org).

