# Supplementary material for: SNPs in FNDC5 (irisin) are associated with obesity and modulation of glucose and lipid metabolism in Saudi subjects
Source: Lipids Health Dis. 2016 Mar 11;15:54. doi: 10.1186/s12944-016-0224-5 (PMC4788945; doi:10.1186/s12944-016-0224-5)
Supplement: Additional file 1: Table S1. — Details of used TaqMan assays, chromosomal location and minor allele frequencies for the studied SNPs. (DOCX 13 kb) [file 12944_2016_224_MOESM1_ESM.docx]

**Supplementary Table 1:** Details of used TaqMan assays, chromosomal location and minor allele frequencies for the studied SNPs

| **SNP ID** | **NCBI Assembly Location** | **TaqMan**  **Assay ID** | **Context Sequence [VIC/FAM]** | **G-MAF** | **S-MAF** |
| --- | --- | --- | --- | --- | --- |
| rs3480 | ch. 1: 33328165 | C___8822841_10 | AGACCGGAAGGAAGGGGCGGTCATT[A/G]GGTGATGGCTTCTGGCTCTCTGGCT | 0.41 | 0.44 |
| rs1746661 | ch. 1: 33335039 | C___8854680_1_ | TGCCCTGTCCAATGAGTGACCTTGC[G/T]CGAAGGAGAAAGACCCAGAGATCGA | 0.14 | 0.23 |
| rs1298190 | ch. 1: 33340038 | C___8854690_10 | GAGGGCTTGATCCAGTCTGCCTACC[A/G]TATCCCCTCACCTCTAAGGACCTCT | 0.17 | 0.13 |
| rs7246334 | ch. 1: 33333104 | C____927694_10 | AGCCCCAAGAAGCTGAACCTCTTCT[A/G]AGGGAGGGCGAAGGCAAGTACTCAT | 0.36 | 0.38 |
| rs1570569 | ch. 1: 33336956 | C___8854681_10 | AGCAGAGGGGAGAGATCACAGTAAG[G/T]CTGGGGTGAGCAGAGAGTGCTAAGT | 0.14 | 0.23 |

G-MAF: Global minor allele frequency (Source – 1000 genomes)

S-MAF: Saudi minor allele frequency (present study)
